# CD1b glycoprotein, a crucial marker of thymocyte development during T cell maturation in cynomolgus monkeys

**DOI:** 10.1038/s41598-023-41708-y

**Published:** 2023-09-01

**Authors:** Sung Min Choi, Hi Jung Park, Eun A Choi, Kyeong Cheon Jung, Jae Il Lee

**Affiliations:** 1 Graduate Course of Translational Medicine, Seoul National University College of Medicine, Seoul, 03080 Republic of Korea; 2 Transplantation Research Institute, Seoul National University College of Medicine, Seoul, 03080 Republic of Korea; 3 Department of Pathology, Seoul National University College of Medicine, Seoul, 03080 Republic of Korea; 4 Integrated Major in Innovative Medical Science, Seoul National University Graduate School, Seoul, 03080 Republic of Korea; 5 Department of Medicine, Seoul National University College of Medicine, Seoul, 03080 Republic of Korea

**Keywords:** Cell biology, Immunology

## Abstract

Phenotypic markers that denote different developmental stages of thymocytes are important for understanding T cell development in the thymus. Here, we show that CD1b is a critical discriminator of thymocyte maturation stage in cynomolgus monkeys. CD1b was expressed by immature thymocytes prior to β-selection, and its expression decreased as cells became fully mature in the thymus. MHC-I expression was lowest at the CD3^lo^CD1b^+^ immature double-positive (DP) stage, while the ratio of CD1d:MHC-I expression was significantly higher at this stage than at other developmental stages. PLZF was expressed by < 0.2% of thymocytes; most PLZF^+^ thymocytes were CD3^-/lo^CD1b^+^ immature DP thymocytes with the potential to produce IL-4. EOMES^+^ thymocytes, which accounted for > 2% of total thymocytes, were mostly CD3^+^CD1b^-^ mature thymocytes and predominantly of the CD8 single-positive (SP) lineage. An unconventional CD8^+^ T cell subset expressing the NKG2AC^+^CXCR3^+^ innate-like T cell marker was identified within the EOMES^+^ CD8 SP lineage; these cells exhibited a memory phenotype. Taken together, these findings show that CD1b is a valuable discriminatory marker of thymocyte development. The data presented herein can be used to characterize the features of PLZF- and EOMES-associated unconventional T cells in the thymus.

## Introduction

The thymus produces multiple distinct subsets of T cells that mature into functionally diverse T cell lineages. However, to better understand the development of conventional or unconventional T cells, specific markers are needed to distinguish between different stages of thymocyte development. The CD1 molecule, which comprises five different isoforms, is a family of transmembrane glycoproteins associated with β2-microglobulin and has a structure similar to major histocompatibility complex (MHC) class I molecules^1^. In humans, CD1a is a known marker of T cell developmental stage in the thymus^2^. Expression of the gene encoding CD1a/c is exceptionally high in tissues of the pig-tailed macaques^3^. However, not all CD1 isoforms are expressed by all species; for example, mice and rats have lost the CD1a, b, c, and e genes while duplicating the CD1d gene^4^. This means that CD1a cannot be used as a distinguishing marker in all species.

Although the amino acid sequence of group 1 CD1 proteins in nonhuman primates is highly conserved^5^, it is not clear whether CD1a is expressed by thymocytes. However, previous research shows that CD1b is expressed by immature CD4^+^CD8^+^ double-positive (DP) thymocytes, allowing them to be distinguished from peripheral CD4^+^CD8^+^ DP T cells^6^. Identifying other surface molecules involved in thymocyte emigration, such as CCR7 and CD69^7^, will help demonstrate that CD1b is a useful marker for distinguishing the thymocyte development stage.

In addition to conventional CD4^+^ or CD8^+^ T cells, a variety of unconventional T cell types have been identified in the periphery of humans^8^ and nonhuman primates^9^ as well as in the transcriptome of the human thymus^10^; however, little is known about the characteristics of these subsets in the thymus of nonhuman primates. PLZF is a key regulator of unconventional T cell development, including iNKT and MAIT cells^11^, while EOMES is a crucial factor for the development of innate-like T cells^12^. However, these transcriptional factors may also be involved in development of other unconventional T cell types. Thorough phenotypic characterization of PLZF^+^ T cells and EOMES^+^ T cell subsets in the thymus is important if we are to better understand their immunological functions; these molecules are also potentially useful biomarkers for monitoring immune responses to vaccination, infection, and disease. Therefore, the aim of this study is to investigate whether CD1b is a crucial surface marker that distinguishes thymocyte developmental stage and, based on this, to further explore involvement of PLZF or EOMES in development of unconventional T cells.

## Results

### CD1b is a crucial marker of thymocyte maturation stage

**Figure 1:**
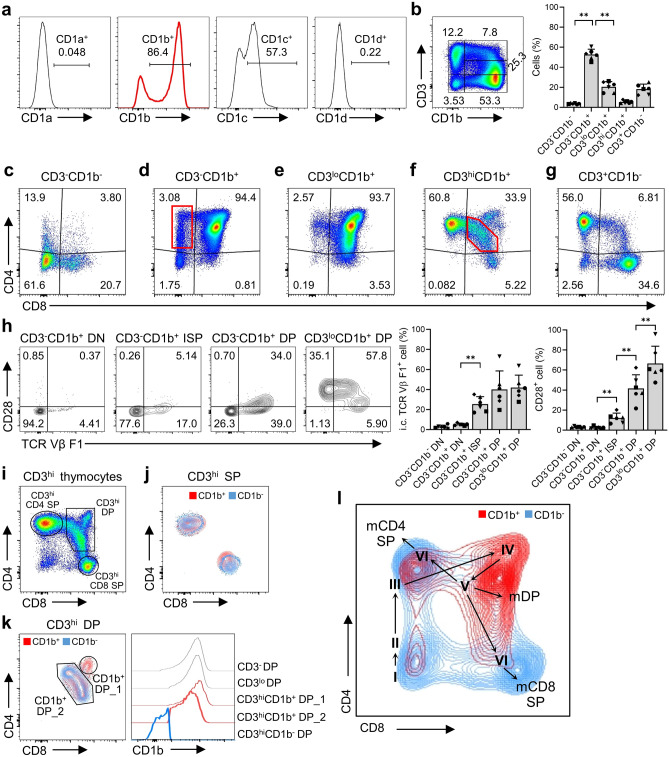
(**a**) Histograms showing expression of CD1a, CD1b, CD1c, and CD1d by total thymocytes. (**b**) Dot plot showing the five developmental stages, defined according to expression of CD3 and CD1b, and a graph showing the percentage of cells at each developmental stage. (**c–g**) Dot plots showing CD4/CD8 expression patterns at each developmental stage. The red box in panel d denotes the ISP stage, and the red box in panel f denotes the CD4^hi^CD8^lo^ intermediate stage. (**h**) Dot plots and graphs showing expression of intracellular TCR Vβ and CD28 according to developmental stage (up to CD3^lo^CD1b^+^ DP stage). (**i**) Dot plots showing CD4/CD8 expression by CD3^hi^ thymocytes. (**j**) A contour plot showing CD1b^+^ and CD1b^−^ thymocytes among CD3^hi^ CD4/CD8 SP thymocytes. (**k**) A contour plot showing CD1b^+^ and CD1b^−^ thymocytes among CD3^hi^ DP thymocytes and a histogram comparing CD1b expression levels by DP thymocytes at each developmental stage. This histogram uses the modal option to scale all channels as a percentage of the maximum count. (**l**) A schematic diagram showing the order of the developmental stages of thymocytes, based on the expression of CD1b. Each number indicates the following stage: (I) CD3^-^CD1b^-^ DN, (II) CD3^-^CD1b^+^ DN, (III) CD3^-^CD1b^+^ ISP, (IV) CD3^-^CD1b^+^ DP and CD3^lo^CD1b^+^ DP, (V) CD3^hi^CD1b^+^ DP, (VI) CD3^hi^CD1b^+^ SP, mCD4 SP: CD3^+^CD1b^-^ mature CD4 SP, mCD8 SP: CD3^+^CD1b^-^ mature CD8 SP, mDP: CD3^+^CD1b^-^ mature DP. Data are representative of six independent experiments, each with a similar result. Data are expressed as the mean ± SD (n = 6). *p < 0.05; **p < 0.01. i.c.: intracellular.

To better understand the development of thymocytes from the CD4/CD8 double-negative (DN) stage to the CD4 or CD8 single-positive (SP) stage, we tried to identify a distinguishable marker. We observed expression of four type-isoforms of group 1 CD1 molecules, with the exception of CD1e, which is only expressed intracellularly. CD1a, which is used as an indicator of human thymic development^2^, was expressed at very low levels by the total thymocyte population of cynomolgus monkeys. Similarly, CD1d was expressed at low levels. Although CD1c was expressed in the thymus at higher levels than CD1a and CD1d, it was confined mainly to CD3^-^ or CD3^lo^ thymocytes and was not nearly as prominent in the CD3^hi^ immature population (Fig. 1a and Supplementary Fig. S1a). Although the expression of CD3, an essential component of the TCR complex, alone can indicate differentiation from DN to DP thymocytes, it is limited in distinguishing the stages of transition (Supplementary Fig. S1b). By contrast, we found that the CD1b molecule was highly and widely expressed by thymocytes, and that it can be used to distinguish between the different stages of thymocyte development (Fig. 1b).

Based on the expression level of CD3 and CD1b, we defined the following five stages of thymocyte maturation from CD4/CD8 DN to CD4 or CD8 SP: CD3^-^CD1b^-^, CD3^-^CD1b^+^, CD3^lo^CD1b^+^, CD3^hi^CD1b^+^, and CD3^+^CD1b^-^ (Fig. 1c–g). At the early developmental stage, CD1b was rarely expressed by CD4/CD8 DN cells, which expressed neither CD4 nor CD8 coreceptors (Fig. 1c). However, as expression of CD1b increased gradually, CD4/CD8 DN thymocytes converted to CD4/CD8 DP thymocytes. During transition of CD3^-^ immature thymocytes from CD1b^-^ to CD1b^+^, we observed the appearance of CD4^+^CD8^-^ immature single-positive (ISP) thymocytes, which preferentially express the CD4 coreceptor (Fig. 1d). This population is identical to CD3^-^CD4^+^CD8^-^ immature thymocytes found in humans^13^. We also observed a significant increase in expression of CD28, a marker of β-selection^14^, along with T cell receptor (TCR)-β, from the ISP stage (Fig. 1h). These TCR signaling-related molecules were expressed after expression of CD1b began, which is consistent with a previous report showing that CD1a is expressed by human thymocytes prior to β-selection^15^. As CD3 expression increased, most CD1b^+^ thymocytes differentiated into immature CD4/CD8 DP thymocytes (Fig. 1e). We then observed that CD3^hi^CD1b^+^ DP thymocytes passed through a temporary CD4^hi^CD8^lo^ intermediate stage, characterized by significant downregulation of the CD8 coreceptor, before eventually differentiating into CD4 or CD8 SP thymocytes (Fig. 1f, g). This finding aligns with a previous report showing that signaled CD3^hi^ DP thymocytes initially terminate CD8 transcription and enter a CD4^hi^CD8^lo^ intermediate stage in which expression of CD8 is reduced temporarily^16^.

Next, we investigated whether CD1b expression can be used to distinguish the late maturation stages of SP or DP thymocytes within the CD3^hi^ population (Fig. 1i). We divided immature and mature thymocytes within each lineage based on CD1b levels in the CD3^hi^ fraction. As expected, the CD4 or CD8 SP cell lineages were clearly divided into two subpopulations based upon their CD1b expression levels (Fig. 1j). CD1b expression was lost during full maturation, when immature DP thymocytes committed to the CD4 or CD8 SP lineages. We also analyzed CD3^hi^ DP thymocytes, dividing them into DP_1 (CD4/CD8 very-high DP) and DP_2 (CD4/CD8 high-intermediate DP) subpopulations. These two subpopulations exhibited distinctly different CD1b expression levels. CD1b expression decreased gradually as the DP_1 population converted into the DP_2 population (Fig. 1k). Although small in number, CD3^+^ DP thymocytes that lost CD1b gave rise to mature DP T cell lineages such as CD3^+^ SP T cells. Taken together, the data show that the expression level of CD1b can be used to define the sequential developmental stages of cynomolgus monkey thymocytes (Fig. 1l).

### CD8αα can be expressed transiently or permanently by thymocytes

**Figure 2:**
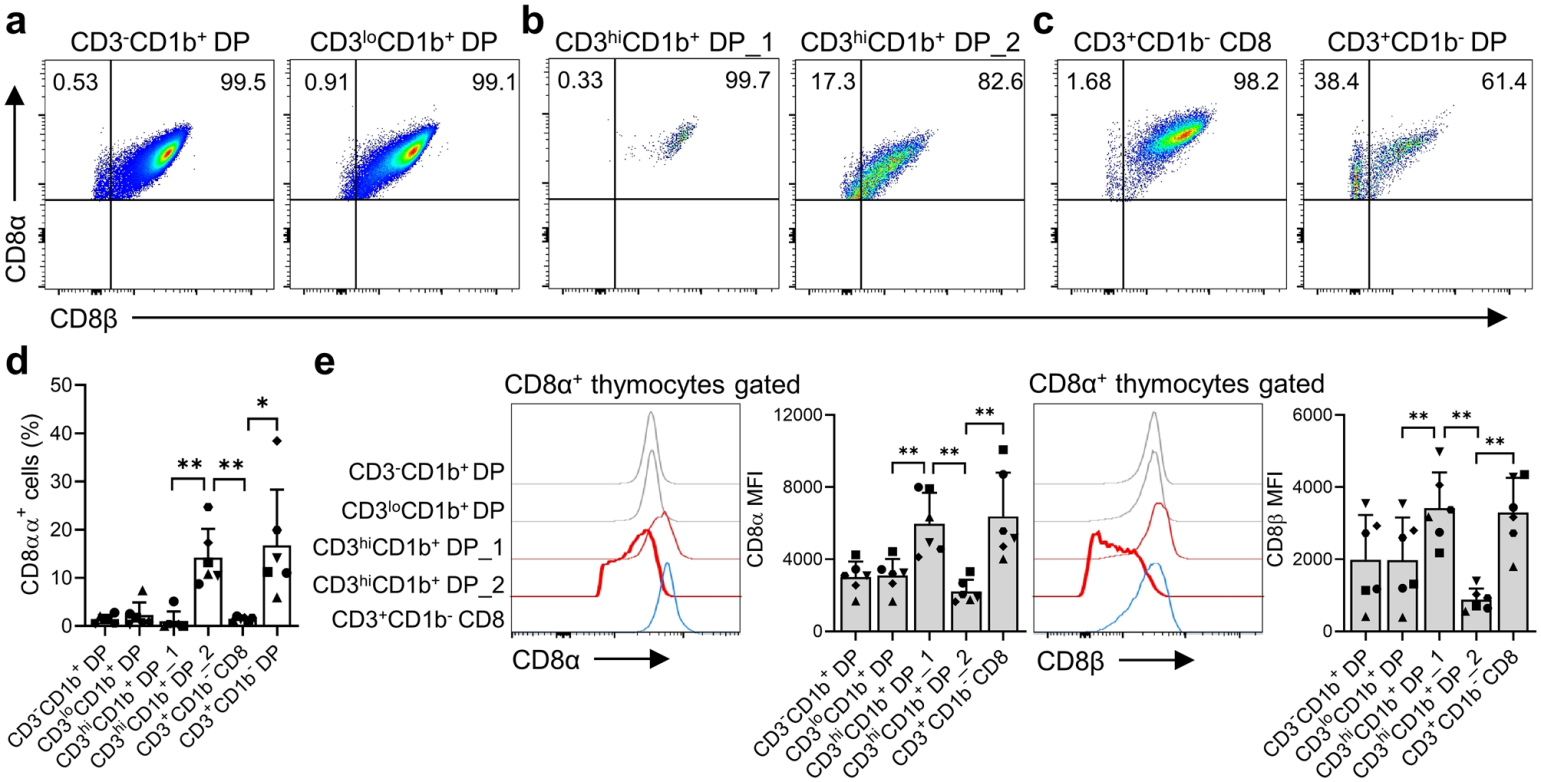
(**a**)–(**c**) Dot plots showing expression of CD8α/β at each developmental stage: (**a**) CD3^-^CD1b^+^ and CD3^lo^CD1b^+^ DP thymocytes, (**b**) CD3^hi^CD1b^+^ DP_1 and DP_2 thymocytes (**c**) CD3^+^CD1b^-^ mature CD8 SP and DP thymocytes. (**d**) A graph showing the percentage of CD8αα-expressing thymocytes at each developmental stage. (**e**) Histograms showing expression levels of CD8α/β and graphs showing the mean fluorescence intensity (MFI) of CD8α/β expression at each developmental stage. Data are representative of six independent experiments, each with a similar result. All histograms use the modal option to scale all channels as a percentage of the maximum count. Data are expressed as the mean ± SD (n = 6). *p < 0.05; **p < 0.01.

To investigate whether CD8αα coreceptors can be induced upon TCR signaling^17^ during thymocytes development, we examined changes in CD8 expression at each developmental stage. We observed the presence of a CD8αα^+^ population, albeit in small numbers, at the CD3^-^CD1b^+^ and CD3^lo^CD1b^+^ developmental stages (Fig. 2a). However, we were unable to define this subset clearly. Additionally, we observed changes in CD8 expression during transition of immature DP thymocytes positively selected at the CD4^hi^CD8^lo^ intermediate stage^16,18^. The results showed a significant reduction in the CD8αβ^+^ population at this stage, while the CD8αα^+^ population increased temporarily by more than 20% (Fig. 2b,d). Interestingly, we found that this decrease in the CD8αβ heterodimer was due to rapid downregulation of the CD8β chain rather than the CD8α chain (Fig. 2e). Furthermore, a significant proportion of CD3^hi^CD1b^+^ immature DP populations that acquired CD8αα homodimer remained as CD3^+^CD1b^-^CD8αα^+^ mature DP thymocytes, while only a few cells were present in the CD8 SP T cell lineages (Fig. 2c,d,e). Taken together, these findings show that CD8αα^+^ cells are present in the monkey thymus from the early developmental stage to the fully mature stage of thymocytes.

### CD31 and CCR7 are expressed most highly just before thymocytes are exported from the thymus

**Figure 3:**
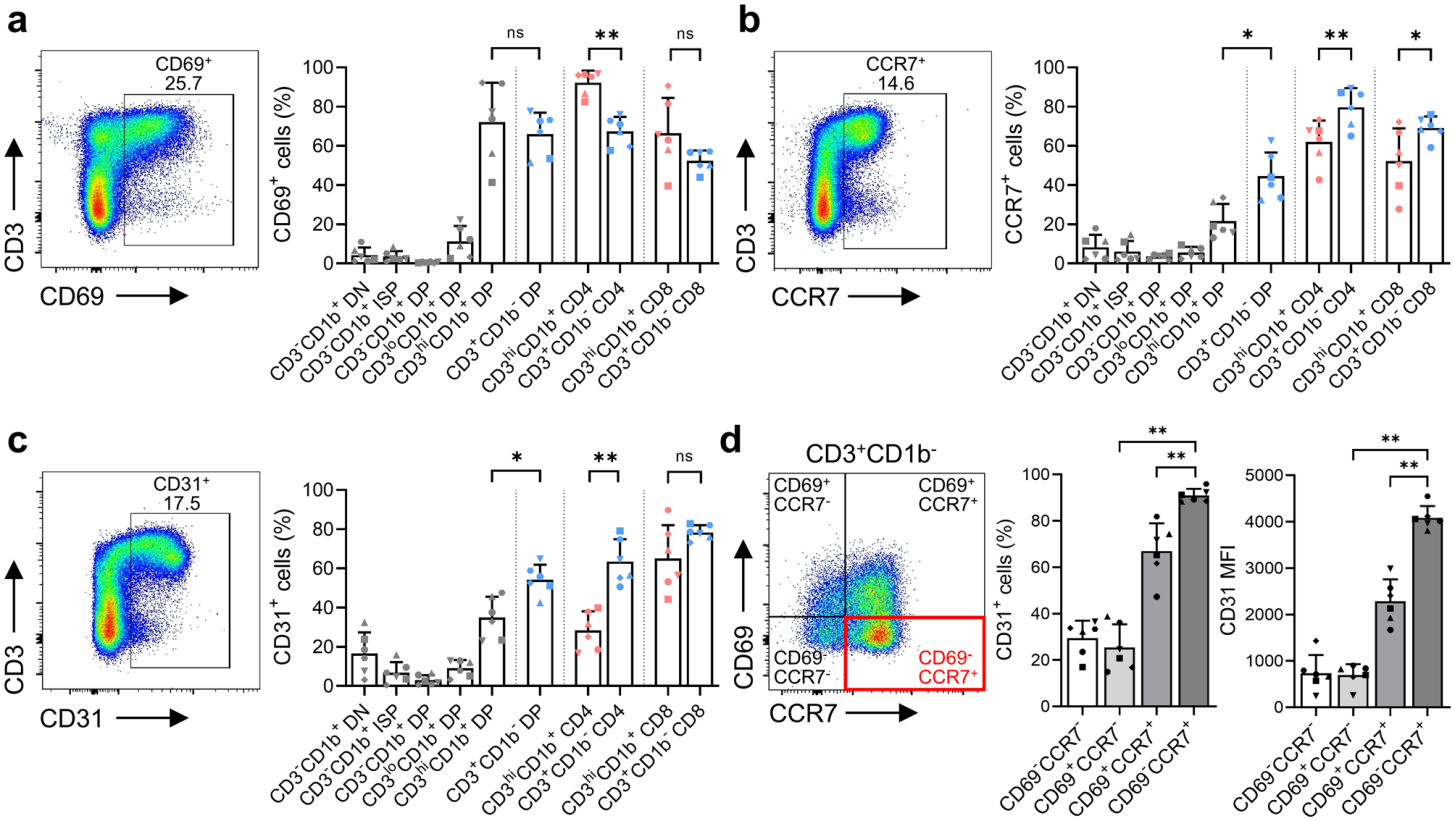
(**a**)–(**c**) Dot plots showing expression of thymic stage markers among total thymocytes, and graphs showing expression of thymic stage markers by each T cell lineage after the CD3^lo^CD1b^+^ DP stage: (**a**) CD69, (**b**) CCR7, and (**c**) CD31. (**d**) A dot plot showing expression of CD69 and CCR7 by CD3^+^CD1b^-^ thymocytes, and graphs showing the percentage of CD31^+^ thymocytes and the mean fluorescence intensity (MFI) of CD31 expression by each population (divided by CD69 and CCR7 expression). Shared developmental stages before CD4/CD8 lineage commitment are represented by gray indicators. Data are representative of six independent experiments, each with similar results. Data are expressed as the mean ± SD (n = 6). *p < 0.05; **p < 0.01; ns: not significant.

To investigate maturation of thymocytes prior to emigration from the thymus, we looked at markers associated with this process. First, we examined CD69, which is expressed transiently by thymocytes undergoing β-selection or positive selection in the thymus^19^. We found that the proportion of CD69^+^ cells in CD3^lo^CD1b^+^ immature DP thymocytes increased gradually, reaching its highest at the CD3^hi^CD1b^+^ immature stage. Subsequently, we observed a marked decrease in CD69^+^ thymocytes, along with loss of CD1b, by the mature SP and DP lineages (Fig. 3a). This transient alteration of CD69 expression by thymocytes is consistent with reports that CD69 plays a potential role in regulating thymocyte export^7,20^. We also examined the expression of CCR7, which is associated with medullary migration^21^. We found that the proportion of CCR7^+^ cells increased from the CD3^hi^CD1b^+^ immature DP stage until the CD3^+^CD1b^-^ mature stage of the SP and DP lineages (Fig. 3b).

Thymocytes expressing CD31, known as a marker of recent thymic emigrants (RTEs) and late-stage thymocytes^22^, was also increased with the thymocyte maturation, and its proportion was highest at the CD3^+^CD1b^-^ mature stage of the SP and DP lineages (Fig. 3c). However, not all mature thymocytes expressing CD31 egress from the thymus. To define RTEs, we compared expression levels of CD31 based on expression of CD69 and CCR7 by CD3^+^CD1b^-^ mature thymocytes. As expected, CD31 expression by the CD69^-^CCR7^+^ fraction was higher than that by the CD69^+^CCR7^+^ fraction, confirming that CD31 can indeed be a phenotypic feature of RTEs (Fig. 3d). Although a very small subset, a population of CD3^+^CD1b^-^CD69^-^CCR7^+^CD31^hi^ cells was clearly identified at the mature DP stage (Supplementary Fig. S1c), indicating that they can emigrate to the peripheral blood as fully mature CD4^+^ or CD8^+^ SP T cell lineages. This finding is consistent with reports showing that the naive DP T cells observed in the peripheral blood originate from these fully mature DP T cells in the thymus^23,24^. Eventually, expression of CD1b and CD69 molecules shows an inverse relationship with that of CCR7 and CD31 before the mature SP and DP lineages are exported from the thymus. These results are consistent with those of previous reports, showing that fully mature SP thymocytes exhibit an RTEs phenotype distinct from most other medullary thymocytes^25,26^. Taken together, the data confirm that the CD69^-^CCR7^+^CD31^hi^ cell population represents a phenotype of RTEs that is distinct from most other medullary CD3^+^CD1b^-^ mature thymocytes in cynomolgus monkeys.

### Expression of MHC-I molecules by immature DP thymocytes is associated with development of unconventional T cells

**Figure 4:**
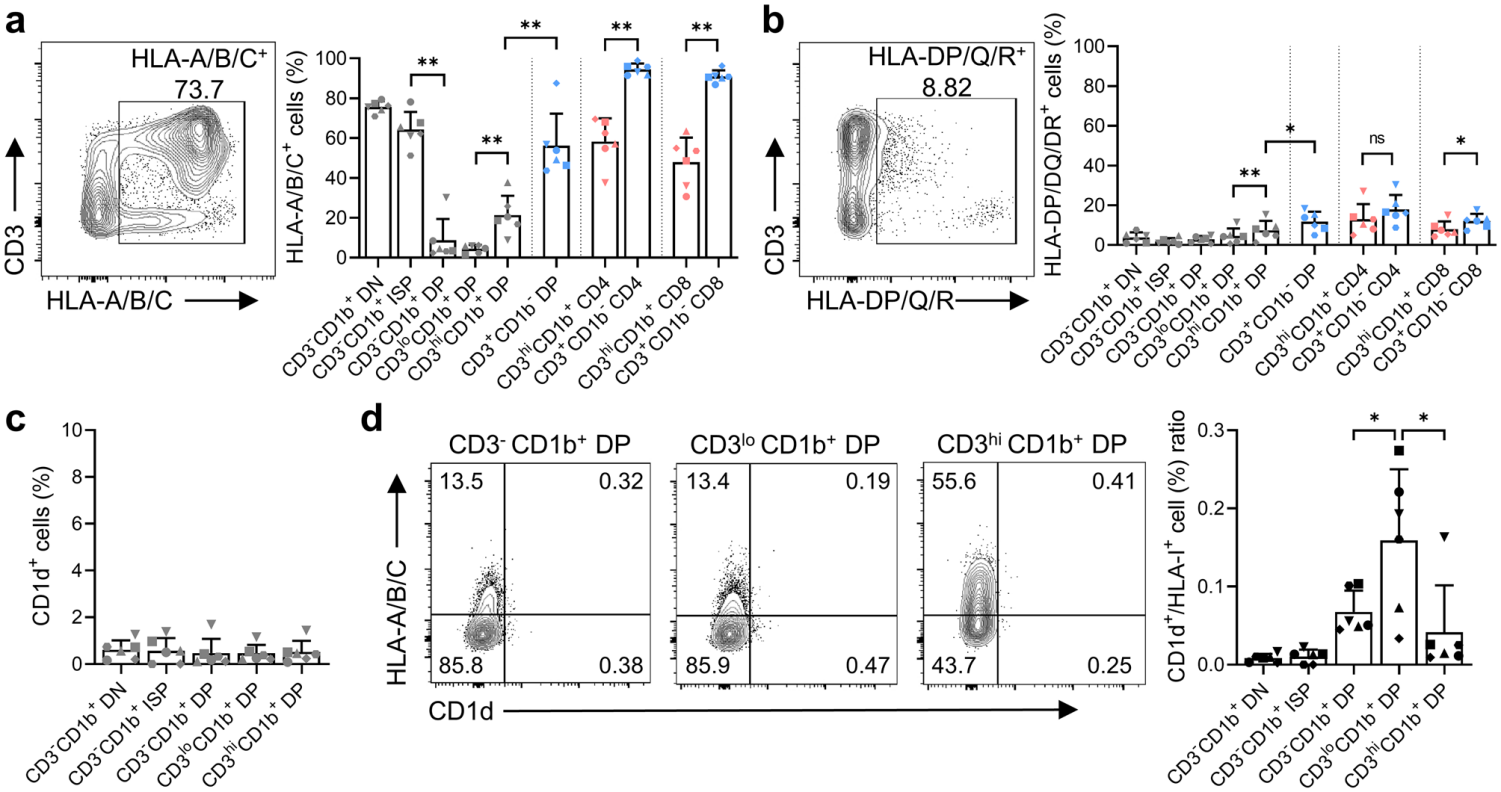
(**a**), (**b**) Dot plots showing expression of MHC-I/II molecules among total thymocytes, and graphs showing expression of MHC-I/II molecules at each developmental stage in each T cell lineage: (**a**) MHC-I (HLA-A/B/C) and (**b**) MHC-II (HLA-DP/DQ/DR). (**c**) Graph showing expression of CD1d at each developmental stage in each T cell lineage. (**d**) Dot plots showing expression of CD1d and MHC-I molecules at each immature DP stage and a graph showing the ratio of CD1d:MHC-I expression according to developmental stage (up to the CD3^hi^ CD1b^+^ DP stage). All analyses of the MHC molecules and CD1d were performed using CD11b^-^CD11c^-^CD14^-^CD20^-^ cells. Shared developmental stages before CD4/CD8 lineage commitment are represented by gray indicators. Data are representative of six independent experiments, each with similar results. All histograms use the modal option to scale all channels as a percentage of the maximum count. Data are expressed as the mean ± SD (n = 6). *p < 0.05; **p < 0.01; ns: not significant.

Thymic selection relies on MHC molecules. Although thymic epithelial cells and thymocytes play a crucial role in development and maturation of T cells^27^, thymocyte-thymocyte interactions also contribute to development of various T cells^28^. To investigate this further, we observed expression of MHC molecules at each developmental stage of thymocyte. We found that expression of MHC-I and MHC-II molecules was not constant throughout the thymocyte maturation process (Fig. 4a,b). Interestingly, thymocytes expressing MHC-I were reduced markedly at the CD3^lo^CD1b^+^ immature DP stage, after which the proportion of MHC-I^+^ thymocytes increased gradually and peaked at the CD3^+^CD1b^-^ mature stage. In contrast with MHC-I molecules, the proportion of MHC-II^+^ thymocytes was not so high in either the SP or DP lineages.

Classical MHC-I molecules expressed by immature DP thymocytes can affect development of unconventional T cells^29^. To better understand the impact of MHC-I molecule expression in the thymus, we analyzed expression of CD1d associated with unconventional T cell development^11^ alongside that of MHC-I. Unexpectedly, we found that the proportion of thymocytes expressing CD1d, an MHC-I like molecule, was low across all developmental stages (Fig. 4c). However, the proportion of MHC-I^+^ thymocytes were significantly low at the CD3^lo^CD1b^+^ immature DP stage, resulting in a higher CD1d^+^:MHC-I^+^ cell ratio than that observed at other stages of maturation (Fig. 4d). These findings support the notion that differential expression of these two molecules by thymocytes likely plays a critical role in development of unconventional T cells.

### PLZF^+^ thymocytes at the immature DP stage express high levels of IL-4

**Figure 5:**
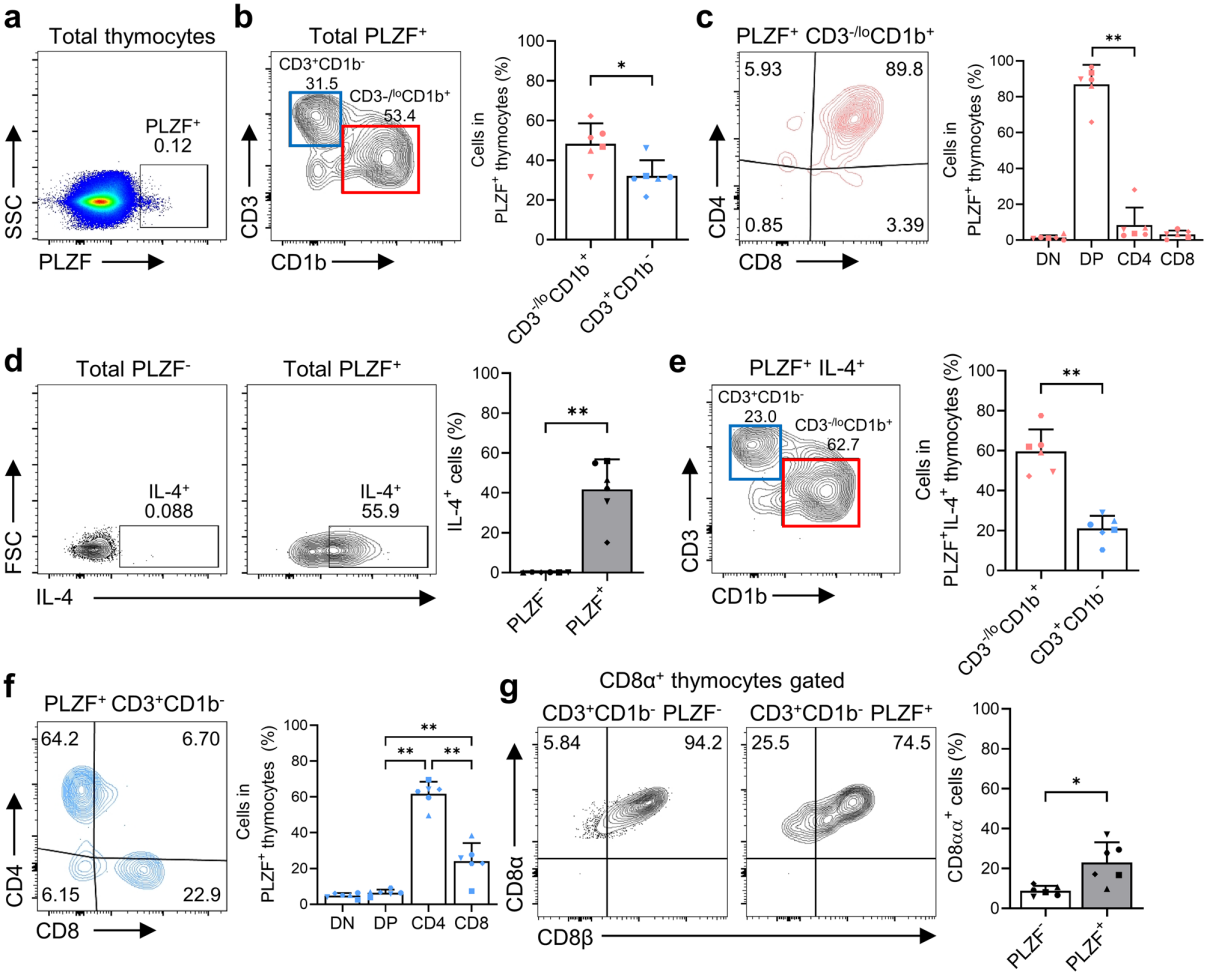
(**a**) A dot plot showing expression of PLZF by total thymocytes. (**b**) A contour plot and a graph showing the developmental stage distribution of PLZF^+^ thymocytes. (**c**) A contour plot and a graph showing CD4/CD8 expression by PLZF^+^ CD3^-/lo^CD1b^+^ thymocytes. (**d**) Contour plots and a graph comparing IL-4^+^ cells within the PLZF^-^ and PLZF^+^ thymocyte populations. (**e**) A contour plot and a graph showing the developmental stage distribution of PLZF^+^IL-4^+^ thymocytes. (**f**) A contour plot and a graph showing CD4/CD8 expression by PLZF^+^ CD3^+^CD1b^-^ thymocytes (excluding Vα24-Jα18^+^ and Vα7.2^+^ thymocytes). (**g**) Contour plots and a graph comparing the percentages of CD8αα^+^ thymocytes within the CD3^+^CD1b^-^ PLZF^-^ and CD3^+^CD1b^-^ PLZF^+^ thymocyte populations (excluding Vα24-Jα18^+^ and Vα7.2^+^ thymocytes). Data are representative of six independent experiments, each with similar results. Data are expressed as the mean ± SD (n = 6). *p < 0.05; **p < 0.01.

To investigate the developmental process of unconventional T cells, we examined expression of PLZF, a transcription factor critical for innate T cell development^30^. The results revealed that less than 0.2% of total thymocytes expressed PLZF (Fig. 5a), with the majority belonging to the CD3^-/lo^CD1b^+^ immature stage, while some were CD3^+^CD1b^-^ mature thymocytes (Fig. 5b). More than 80% of PLZF^+^ thymocytes at the CD3^-/lo^CD1b^+^ DP stage were immature DP thymocytes (Fig. 5c). Given that most NKT cells expressing PLZF branch off from conventional T cell development at the CD4^+^CD8^+^ DP stage^11^, the CD3^-/lo^CD1b^+^ immature stage suggests that DP thymocytes commit to the NKT cell lineage via TCR engagement with CD1d. Moreover, we observed that PLZF^+^ thymocytes at the CD3^-/lo^CD1b^+^ immature DP stage showed greater potential to produce IL-4 than PLZF^-^ thymocytes (Fig. 5d, e).

Next, we focused on a small subset of PLZF^+^ cells at the CD3^+^CD1b^-^ mature stage to better understand their characteristics. Initially, we evaluated PLZF expression in Vα24-Jα18^+^ (NKT) and Vα7.2^+^ (MAIT), and γδTCR^+^ thymocytes (γδT). As expected, PLZF^+^ populations were observed in Vα24-Jα18^+^ and Vα7.2^+^ thymocytes, while γδTCR^+^ thymocytes exhibited very rare PLZF expression (Supplementary Fig. S2a). We found that thymocytes expressing PLZF were present mainly in the CD4 SP lineage, followed by the CD8 SP lineage (Fig. 5f). In addition, a small proportion of PLZF-expressing cells were observed in the CD4^+^CD8^+^ DP thymocyte population at the mature stage. The finding that mature PLZF^+^ CD4 cell subsets are present in the thymus is significant because PLZF is typically expressed by iNKT cells and MAIT cells^11^. This suggests that the presence of these subsets may be important for development of other unconventional T cells. Additionally, more PLZF^+^ cells than PLZF^-^ cells expressed CD8αα (Fig. 5g). Taken together, these findings suggest that PLZF is expressed predominantly at the immature DP stage by thymocytes with the potential to produce IL-4, although expression by mature CD4 or CD8 SP is also clearly observed.

### NKG2AC^+^CD8^+^ thymocytes exhibit an EOMES^+^ memory phenotype

**Figure 6:**
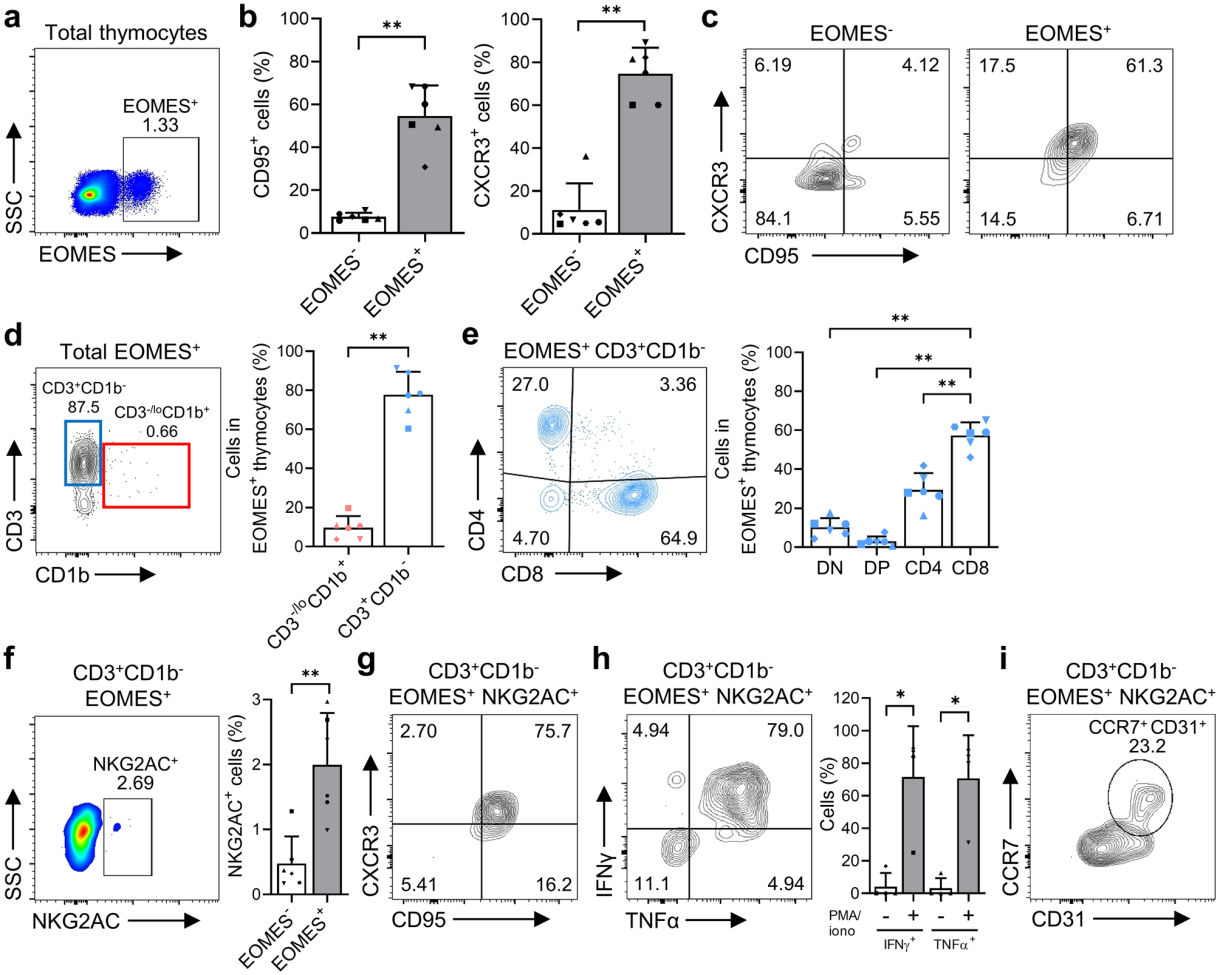
(**a**) A dot plot showing EOMES expression by total thymocytes. (**b, c**) Graphs (**b**) and contour plots (**c**) showing expression of CD95 and CXCR3 by EOMES^-^ and EOMES^+^ thymocytes. (**d**) A contour plot and a graph showing the developmental stage distribution of EOMES^+^ thymocytes. (**e**) A contour plot and a graph showing CD4/CD8 expression of EOEMS^+^ CD3^+^CD1b^-^ thymocytes (excluding Vα24-Jα18^+^ and Vα7.2^+^ thymocytes). (**f**) A dot plot showing NKG2AC^+^ thymocytes within the CD3^+^CD1b^-^ EOMES^+^ thymocyte populations and a graph comparing the percentages of NKG2AC^+^ thymocytes within the EOMES^+^ and EOMES^-^ thymocyte subpopulations within the CD3^+^CD1b^-^ thymocytes population (excluding Vα24-Jα18^+^ and Vα7.2^+^ thymocytes). (**g**) A contour plot showing expression of CD95 and CXCR3 by CD3^+^CD1b^-^ EOMES^+^NKG2AC^+^ thymocytes (excluding Vα24-Jα18^+^ and Vα7.2^+^ thymocytes). (**h**) A contour plot and a graph showing the production of IFNγ and TNFα by CD3^+^CD1b^-^ EOMES^+^NKG2AC^+^ thymocytes (excluding Vα24-Jα18^+^ and Vα7.2^+^ thymocytes) after PMA/iono stimulation (n = 4). (**i**) A contour plot showing expression of CCR7 and CD31 by CD3^+^CD1b^-^ EOMES^+^NKG2AC^+^ thymocytes (excluding Vα24-Jα18^+^ and Vα7.2^+^ thymocytes). Data are representative of six independent experiments, each with similar results. Data are expressed as the mean ± SD (n = 6). *p < 0.05; **p < 0.01.

In this study, we found that over 2% of thymocytes express EOMES (Fig. 6a), similar to previous reports^31^. Most of these EOMES^+^ cells had a CD95^+^ memory phenotype and expressed CXCR3, a marker of innate-memory CD8 T cells^32^ (Fig. 6b,c). Unlike PLZF^+^ thymocytes, which were found mainly at the CD3^-/lo^CD1b^+^ immature stage, EOMES^+^ thymocytes were present mostly at the CD3^+^CD1b^-^ mature stage (Fig. 6d). The CD8 SP lineage constituted the majority of the population among the CD3^+^CD1b^-^ mature EOMES^+^ population, but the proportion of the CD4 SP lineage was also relatively high (Fig. 6e).

A small number of EOMES^+^ innate-like T cells with a memory phenotype have been identified, in human umbilical cord blood or peripheral blood^33^. Therefore, we asked whether a similar unconventional T cell lineage exists in the thymus. We found a small subset of EOMES^+^ mature thymocytes expressing NKG2AC (Fig. 6f) and observed that these cells coexpressed CD95 and CXCR3 (Fig. 6g). Interestingly, we found that the CD8αα^+^ population was significantly larger in both EOMES^+^ thymocytes and EOMES^+^NKG2AC^+^ thymocytes compared to the EOMES^-^ population (Supplementary Fig.S2b). To validate the functionality of EOMES^+^ innate-like cells, we investigated their cytokine secretion capacity in response to PMA/Iono stimulation in both the EOMES^+^ and EOMES^+^NKG2AC^+^ subsets. The EOMES^+^ subset displayed a higher secretion capacity for IFN-γ and TNF-α compared to the EOMES^-^ population (Supplementary Fig. S2c). Additionally, the EOMES^+^NKG2AC^+^ subset exhibited an even more robust secretion capacity, indicating its potential for innate-like functionality in response to stimuli (Fig. 6h). Furthermore, we investigated recent markers of thymic emigrants to determine if this subset can be exported from the thymus. We found that some EOMES^+^NKG2AC^+^ cells at the CD3^+^CD1b^-^ mature thymocyte stage expressed CCR7 and CD31, although this population was small (Fig. 6i). Therefore, the data suggest that EOMES^+^NKG2AC^+^ memory-like CD8 SP thymocytes expressing CD8αα are a distinct subset of unconventional T cells in the thymus. Moreover, this subset differs from the CD8αα^+^EOMES^+^ CD8 T cells with an effector or terminally differentiated memory phenotype observed in peripheral blood^34^ in terms of expression of recent thymic markers.

## Discussion

By analyzing expression of CD1b and CD3, we were able to discern the developmental stages of thymocytes. This phenotypic approach enabled precise identification of molecular markers associated with thymic selection and various T cell lineages at each developmental stage. The analysis solely based on CD3 expression was insufficient to clearly distinguish between the ISP stage, representing the transitional stage, and the mature stage of DP thymocytes. However, when compared to previously used markers such as CD28, TCRβ, CD31, CD69, and CCR7, the gating strategy based on CD3 and CD1b molecules was able to clearly indicate the five-step differentiation process of T-cell development. The observation of a CD8αα^+^ population at the CD3^-^CD1b^-^ stage (Fig. 1c) before β-selection was intriguing. Although we could not clearly define this population, it seems to represent the early DP population undergoing proliferation, as there is no CD8 expression in the APCs (e.g., DC, macrophage, and B cells) of cynomolgus monkeys^35^, and APCs were excluded before analysis (Supplementary Fig. S3). Certain anti-CD1a clones have been used to identify thymocytes in macaques^5, 36^, however, the expression levels of CD1a observed in our study were not high enough to clearly distinguish developmental stages. Downregulation of CD1a expression by developing T cells correlates with functional maturation, which includes sustained interactions between MHC and TCR molecules^2^. Consistent with this, we found that the CD1b molecule in cynomolgus monkeys displayed a dynamic expression pattern similar to that of CD1a in the human thymus.

Although we did not fully define the role of CD1b expression by thymocytes in this study, it is possible that CD1b molecules play a role in development of T cell subsets that recognize nonprotein antigens such as lipids, glycolipids, and lipopeptides presented by group 1 CD1 molecules (CD1a, CD1b, and CD1c)^37,38^. Therefore, CD1b molecules may promote development of various T cell lineages that recognize lipid antigens through interactions between thymocytes. In humans, studies of the polyclonal CD1-restricted T cell repertoire and ex vivo frequency analysis of CD1-reactive T cell clones reveal that CD1a- and CD1c- autoreactive T cells are detected most frequently; indeed, they may comprise up to 10% of peripheral blood T cells^39^. Additionally, it is worth noting that there are distinct T cell subsets in the periphery that specifically recognize nonprotein antigens in a TCR-dependent manner^5^. These subsets include αβT cells that recognize lipid, glycolipid, and lipopeptide antigens presented by group 1 CD1 molecules and are involved in host defense against mycobacterial infection^40^.

Regarding the transient increase in the CD8αα^+^ population at the CD3^hi^CD1b^+^ DP stage, our findings support the notion that CD8αα can be induced upon activation via the TCR-CD3 complex, and that the degree of induction increases proportionally with signal strength^17,41^. CD8αα acquired at this stage can be expressed transiently or permanently by peripheral T cells^41^ and may serve as an effective TCR coreceptor for avidity enhancement rather than functional signaling. However, the presence of CD8αα^+^ populations at the CD3^-^CD1b^+^ and CD3^lo^CD1b^+^ DP stages has significant implications with respect to affinity for self-antigens^42^. That is, some preselected thymocytes (i.e., prior to conventional selection) express CD8αα and temporarily reduce the TCR signal strength by sequestering Lck and LAT from CD8αβ^41^, leading to survival of agonist-selected thymocytes. These CD8αα^+^ T develop during thymic selection and are present in the peripheral blood of children as well as in cord blood^42^. Although we cannot determine whether they are CD4^+^CD8αα^+^CD8αβ^+^ triple-positive T cells, as observed in the agonist selection pathwy^41–43^, we presume that CD8αα coreceptor expression is deeply involved in alternative selection of CD3^-/lo^CD1b^+^ immature thymocytes, which adopt distinct functional fates. Our observations, therefore, indicate that a variety of T cells characterized by CD8αα expression can arise in cynomolgus monkeys through thymic selection.

DP thymocytes express nonclassical MHC molecules CD1d and MR1, which present nonpeptides and promote selection of iNKT and MAIT cells, respectively, expressing PLZF^44^. A recent study reports that expression of classical MHC by DP thymocytes impairs selection of nonclassical MHC-restricted innate-like T cells^29^. This implies that unconventional T cells develop when expression of classical MHC molecules is reduced, and interaction with nonclassical MHC molecules is increased. In this respect, our results demonstrate that transient and drastic reduction in expression of MHC-I molecules is consistent with the physiological alterations necessary for development of PLZF^+^ innate-like T cells such as iNKT and MAIT. Furthermore, the presence of PLZF^+^ thymocytes at the mature stage (with the exception of iNKT and MAIT cells) suggests that development of other PLZF^+^-related T cells may also occur in cynomolgus monkeys; such cells may include the PLZF^+^ NKT-like cells and PLZF^+^ Th17-like cells found in the human thymus^10^. Our results also indicate that PLZF^+^/IL-4^+^ thymocytes are enriched at the immature DP stage and can be potential sources of a variety of unconventional T cells such as PLZF^+^ innate-like (PIL)^29^ or iNKT2^45^ cells. Furthermore, the presence of PLZF^+^ cells expressing CD8αα within the mature thymocyte population may be associated with development of various T cell lineages, including CD8αα^+^ T cells in the thymus^10^ or PLZF^+^CD8αα^+^ T cells in the peripheral blood^8^. We excluded NKT and MAIT cells using canonical TCR markers. However, it is important to note that these subsets can also express TCRs other than Vα24-Jα18 and Vα7.2, which resulted in a few remaining populations. Hence, we recognize the limitation in achieving complete exclusion of these subsets.

The EOMES^+^ memory-like population is induced by PLZF^+^ cells, which are the major source of IL-4 in the thymus^46^. Our findings show that IL-4^+^ cells within the CD3^-/lo^CD1b^+^ immature thymocyte population were PLZF^+^, indicating that the presence of a PLZF^+^IL-4^+^ cell subset may provide an environment that supports development of an EOMES^+^ memory-like cell subset^46^. A previous study shows that murine EOMES^+^ memory-phenotype CD8^+^ T cells, which react with self-ligands, are derived from T cell precursors expressing the CD8 memory-phenotype TCR, and that they upregulate EOMES during maturation in the thymus^47^. Moreover, recent studies show that NKG2AC^+^CD8^+^ T cells, which are innate-memory cells from rhCMV- or SIV-infected macaques, help to control viral infection by increasing expression of EOMES and production of IFN-γ via IL-15 stimulation^9^. Although the innate-like function of this cell subset was not investigated, we identified for the first time the existence of EOMES^+^NKG2AC^+^ CD8^+^ T cells with an innate-like memory phenotype in the thymus. We further suggest that these EOMES^+^ memory-phenotype CD8 T cells may display activated characteristics as well as innate-like functional properties in the periphery.

Since cynomolgus monkeys are the phylogenetically closest relatives of humans, they may recapitulate immune responses comparable with those observed in humans. Therefore, it is crucial to define molecular markers of T cell development in the thymus and to identify development of other unconventional T cells. Here, we show the importance of the glycoprotein CD1b as a discriminator of thymocyte maturation stage and how its expression levels change during thymocyte development. The findings related to expression of PLZF and EOMES at different T cell lineages, including identification of an unconventional CD8^+^ T cell subset, provide additional insight into the complexity of T cell development in the thymus.

## Materials and methods

### Subjects

Six healthy male cynomolgus monkeys (*Macaca fascicularis*), aged 2–3 years, were used in this study. All animals were cared for in strict accordance with the National Institutes of Health Guide for the Care and Use of Laboratory Animals, and the study was approved by the local Institutional Animal Care and Use Committee (IACUC) of Seoul National University Hospital (IACUC number: 21-0297-S1A0). All experiments were conducted in accordance with relevant guidelines and regulations, including the ARRIVE guidelines.

### Cell preparation

The monkeys were first deeply anesthetized by an intramuscular injection of ketamine (10 mg/kg, Yuhan, Korea) and an intravenous injection of sodium pentobarbital (25 mg/kg, Hanlim Pharm. Co. Ltd, Korea), and subsequently euthanized by exsanguination. All tissues were processed immediately after isolation. Thymus tissues were minced into a single-cell suspension using a 70 μm filter and a syringe plunger. The procedure was repeated until the tissue was completely dissociated. The dissociated cells were resuspended in RPMI 1640 (Biowest, Nuaillé, France) supplemented with 10% fetal bovine serum (FBS; Biowest). All isolated cells were either used immediately or cryopreserved for future experiments. The results obtained using cells immediately after isolation were considered representative. When cryopreserved cells were used for experiments, thawed cells were resuspended with RPMI 1640 supplemented with 10% FBS, placed in a 96-well round-bottom plate, and cultured in a 37°C CO_2_ incubator for 2–4 h to stabilize and recover.

### Antibodies

The following fluorochrome-labeled human monoclonal antibodies were used for flow cytometry analysis: CD3-PerCP-Cy™5.5 (SP34-2, BD Biosciences), CD4-PE/Cyanine7 (OKT4, BioLegend), CD8α-Brilliant Violet 711™ (SK1, BioLegend), CD8β- PE/Cyanine7 (SIDI8BEE, eBioscience), CD1a-FITC (NA1/34-HLK, Invitrogen), CD1b-APC (SN13, BioLegend), CD1c-APC (L161, BioLegend), CD1d-Brilliant Violet 421™ (51.1, BioLegend), CD11b-PE (ICRF44, BD Biosciences), CD11c-PE (3.9, BioLegend), CD14-PE (M5E2, BD Biosciences), CD20-PE (2H7, BioLegend), CD28-PE (CD28.2, BD Biosciences), CD31-Brilliant Violet 421™ (WM59, BioLegend), CD69 (FN50, BioLegend), HLA-ABC (G46-2.6, BD Biosciences), HLA-DR, DP, DQ (Tü39, BioLegend), CCR7-PE (G043H7, BioLegend), CXCR3-PE/Cyanine7 (G025H7, BioLegend), CD95-Brilliant Violet 510™ (DX2, BioLegend), CD159a (NKG2AC)-APC (Z199, Beckman Coulter), TCR Vβ F1- PE/Cyanine7 (8AC, Invitrogen), TCR Vα24-Jα18- APC/Cyanine7 (6B11, BioLegend), TCR Vα7.2-APC/Cyanine7 (3C10, BioLegend), PLZF-PE (Mags.21F7, eBioscience), EOMES-FITC (WD1928, eBioscience), and IL-4-Brilliant Violet 421™ (MP4-25D2, BioLegend).

### Flow cytometry analysis

Prior to surface and intracellular staining, Zombie NIR™ Fixable Viability was used to exclude dead cells. For surface staining, prepared cells were resuspended in staining buffer (PBS, 0.5% BSA, and 0.5 mM EDTA), and single-cell suspensions were labeled with antibodies for 30 min at 4 °C. After surface staining, the cells were washed and resuspended in staining buffer. For intracellular staining, the surface-stained cells were washed with PBS before fixation and permeabilization using the FoxP3/Transcription factor staining buffer set (eBioscience). Then, intracellular cytokines and/or transcription factors were labeled with antibodies for 30 min at 4 °C. Flow cytometry was performed using an LSRFortessa X-20 (BD Biosciences) or LSRII (BD Biosciences) cytometer. All data were analyzed using the FlowJo software v10 (TreeStar, San Carlos, CA, USA).

### Cell stimulation and intracellular cytokine staining

To determine the cytokine production capacity, total thymocytes were resuspended in RPMI 1640 supplemented with 10% FBS and seeded in a 96-well round-bottom plate (5 × 10^5^/well). The cells were then stimulated with 20 ng/ml phorbol 12-myristate 13-acetate (PMA, Sigma-Aldrich, St. Louis, MO, USA) and 2 μg/ml ionomycin (iono, Sigma-Aldrich) for 4 h at 37 °C in the presence of 5 μg/ml brefeldin A (Sigma-Aldrich). For flow cytometry analysis, the stimulated thymocytes were sequentially stained for surface markers and intracellular cytokines, as described above.

### Statistical analysis

Statistical analysis was conducted using the Prism program (GraphPad Software, Inc., USA). Unpaired *t*-tests were used to determine the statistical significance of all analytical data, and a *p*-value < 0.05 was considered significant. The tests performed for each figure are indicated in their respective legends.

### Supplementary Information

Supplementary Figures.

## Data Availability

All data generated or analyzed during this study are included in this published article and its supplementary information files.
