# Supplementary material for: CD1b glycoprotein, a crucial marker of thymocyte development during T cell maturation in cynomolgus monkeys
Source: Sci Rep. 2023 Sep 1;13:14388. doi: 10.1038/s41598-023-41708-y (PMC10474046; doi:10.1038/s41598-023-41708-y)
Supplement: Supplementary file 1 — Supplementary Figures. [file 41598_2023_41708_MOESM1_ESM.pdf]

CD1b glycoprotein, a crucial marker of thymocyte development during T cell maturation in cynomolgus monkeys

Sung Min Choi[1], Hi Jung Park[1], Eun A Choi[1], Kyeong Cheon Jung[2,3,4], and Jae Il Lee[2,5,*]

Supplementary Figure

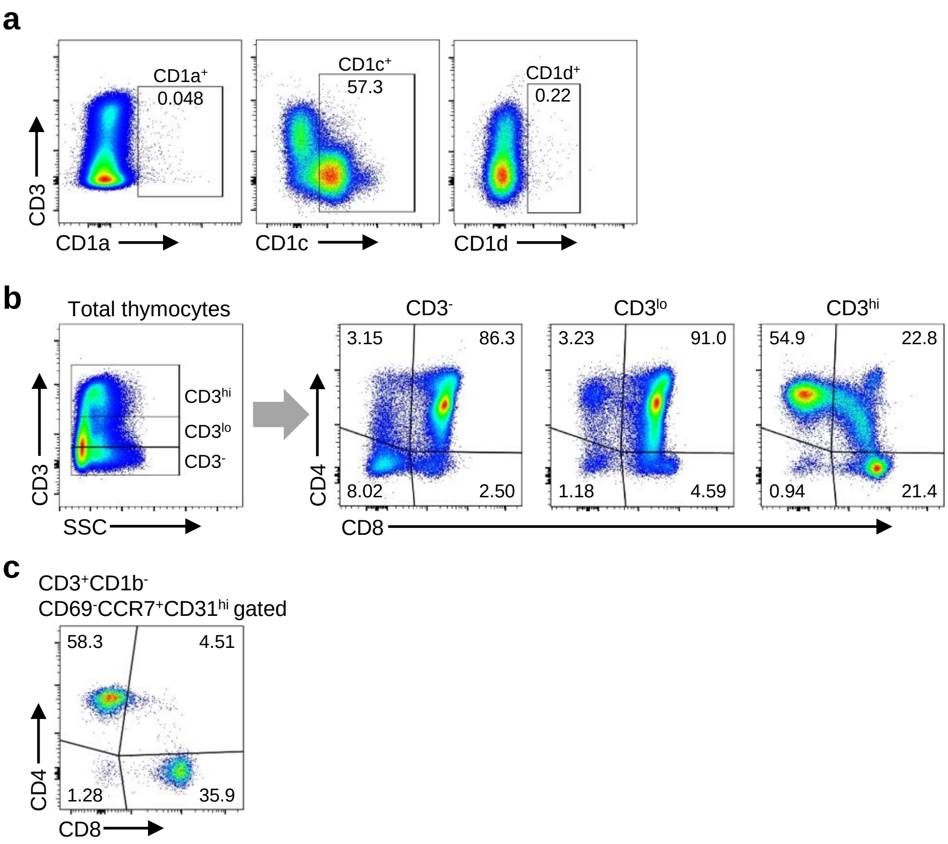

Supplementary figure 1. (**a**) Dot plots showing expression of CD1a, CD1c, and CD1d by the total thymocyte population. (**b**) Dot plots showing developmental stage of thymocytes using CD4/CD8 expression patterns at $CD3^{-}/CD3^{lo}/CD3^{hi}$ thymocytes. (**c**) A dot plot showing CD4 SP, CD8 SP, and DP thymocytes with the RTE phenotype ($CD69^{-}CCR7^{+}CD31^{hi}$) among the fully mature thymocyte ($CD3^{+}CD1b^{-}$) population.

Supplementary Figure

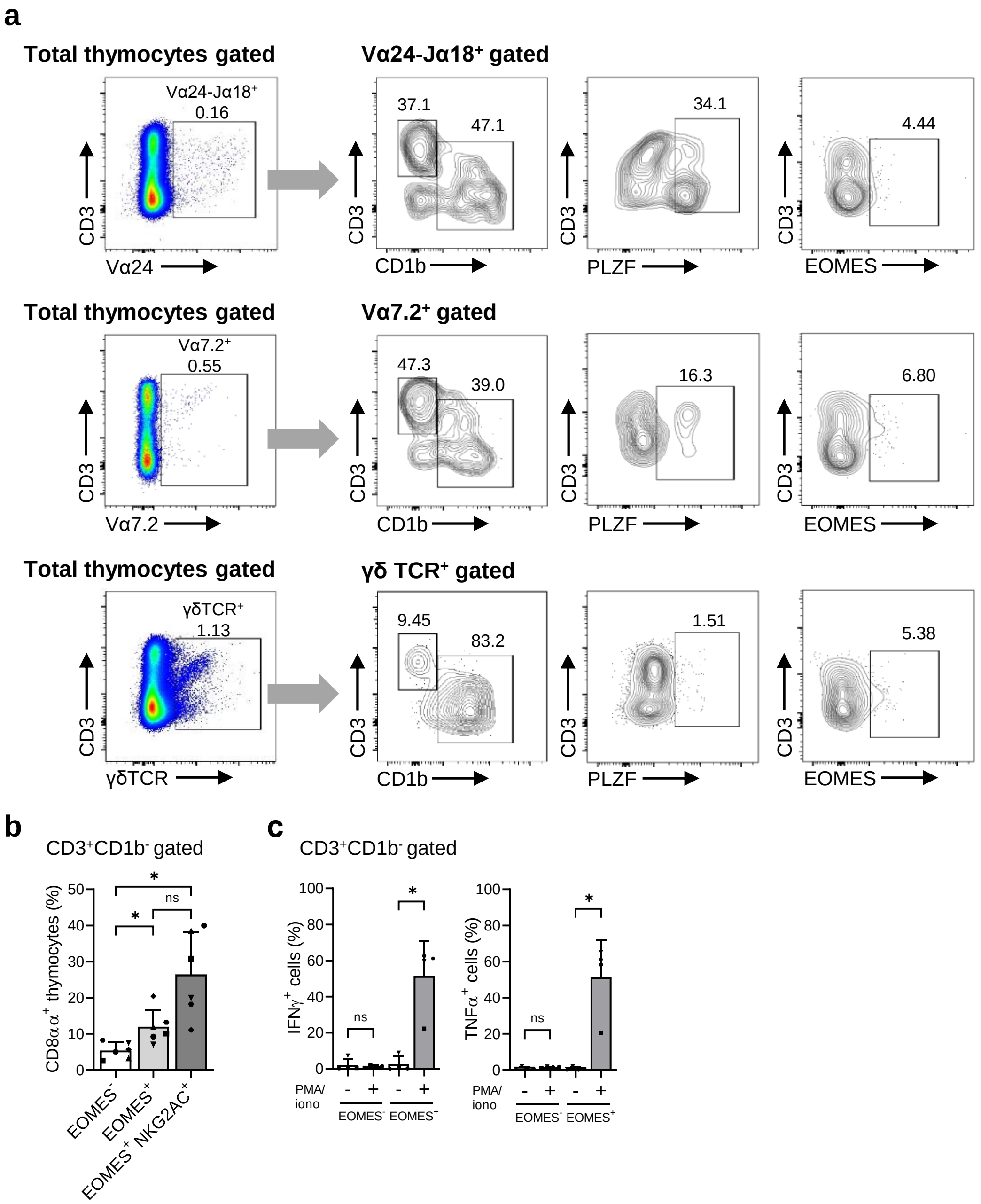

Supplementary figure 2. (**a**) Dot plots showing innate thymocytes (Vα24-Jα18⁺, Vα7.2⁺, and γδTCR⁺ thymocytes) among total thymocytes, and contour plots showing the developmental stage distribution and PLZF/EOMES-expressing populations at each innate thymocytes. (**b**) A graph showing the percentage of CD8αα+ thymocytes at each population: CD3⁺CD1b⁻ EOMES⁻, CD3⁺CD1b⁻ EOMES⁺, and CD3⁺CD1b⁻ EOMES⁺NKG2AC⁺ thymocytes (excluding Vα24-Jα18⁺ and Vα7.2⁺ thymocytes). (**c**) Graphs comparing the production of IFNγ and TNFα by CD3⁺CD1b⁻ EOMES⁻ and CD3⁺CD1b⁻ EOMES⁺ thymocytes (excluding Vα24-Jα18⁺ and Vα7.2⁺ thymocytes) after PMA/iono stimulation (n=4). Data are expressed as the mean ± SD (n = 6). *p < 0.05; **p < 0.01; ns: not significant.

Supplementary Figure

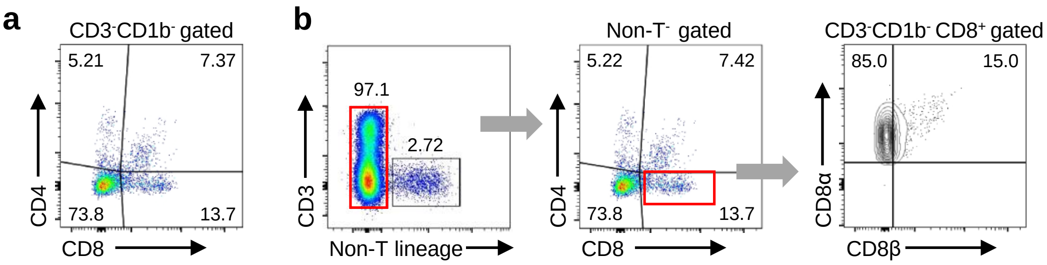

Supplementary figure 3. (a) A dot plot showing $CD3^{-}CD1b^{-}$ stage without the exclusion of antigen-presenting cells (APCs). (**b**) Dot plots showing expression of CD8α/β at $CD3^{-}$ $CD1b^{-}$ $CD8^{+}$ thymocytes after excluding APCs (Non-T lineage markers: $CD11b^{+}$/ $CD11c^{+}$ / $CD14^{+}$ / $CD20^{+}$ cells).
